# Emotional Labor and Burnout of Public Health Nurses during the COVID-19 Pandemic: Mediating Effects of Perceived Health Status and Perceived Organizational Support

**DOI:** 10.3390/ijerph19010549

**Published:** 2022-01-04

**Authors:** Mi-Na Kim, Yang-Sook Yoo, Ok-Hee Cho, Kyung-Hye Hwang

**Affiliations:** 1 College of Nursing, The Catholic University of Korea, 222 Banpo-daero, Seoul 06591, Korea; mina0650@hanmail.net; 2 Department of Nursing, College of Nursing and Health, Kongju National University, 56 Gongju-deahak-ro, Gongju-si 32588, Korea; ohcho@kongju.ac.kr; 3 Department of Nursing, Suwon Science College, 288 Seja-ro, Hwaseong-si 18516, Korea; hkh@ssc.ac.kr

**Keywords:** nurses, public health, emotions, burnout, health status, social support

## Abstract

The purpose of this study was to identify the mediating effects of perceived health status (PHS) and perceived organizational support (POS) in the association between emotional labor and burnout in public health nurses (PHNs). The participants were 207 PHNs convenience sampled from 30 public health centers and offices in Jeju, Korea. Data regarding emotional labor, PHS, POS, and burnout were collected between February and March 2021 using a structured questionnaire. Collected data were analyzed by Pearson’s correlation coefficient and multiple regression analysis. Burnout of PHNs was positively correlated with emotional labor (r = 0.64, *p* < 0.001) and negatively correlated with PHS (r = −0.51, *p* < 0.001) and POS (r = −0.51, *p* < 0.001). In the association between emotional labor and burnout, PHS (B = −1.36, *p* < 0.001) and POS (B = −0.42, *p* = 0.001) had a partial mediating effect. Reduction of burnout among PHNs requires not only effective management of emotional labor but also personal and organizational efforts to improve PHS and POS.

## 1. Introduction

### 1.1. Necessity of Study

The entire world is facing an unprecedented event due to the sudden COVID-19 pandemic. As the pandemic has lingered on and expanded to communities, the public health policies of the government and health authorities are changing constantly. Public health centers are basic but core institutions within the public healthcare network, while public health nurses (PHNs) provide primary healthcare services to community residents for disease prevention and health promotion [1]. However, during the global pandemic crisis, PHNs have faced a sharp increase in their workload for infection control and dealing with complaints [2], screening [3], as well as added work for cohort isolation, contact tracing, and self-isolation training; vaccination; and follow-up actions for adverse symptoms after vaccination [4]. Accordingly, PHNs are suffering from physical and mental fatigue and burnout due to increased workload, emergency shifts, task shifting, and role changes caused by the pandemic [5]. An online survey conducted in the US during August–September 2020 reported that 66.2% of 225 public health workers experienced burnout [6]. The number of Korean PHNs switching jobs during the calendar year 2020 increased by approximately 1.5 times compared to the previous three years [7].

Burnout is a negative psychological experience from repeated exposure to stressors, and for nurses, it is caused or worsened by emotional frustration, lack of competency [8], feeling pushed beyond training [9], emotional labor [10], and lack of organizational support [11]. A previous study that conducted a meta-analysis on prospective studies [12] reported that job burnout is associated with musculoskeletal symptoms, chronic disease, and cerebrovascular disease, and it may cause insomnia, depression, and lower job satisfaction. Burnout of PHNs during the COVID-19 pandemic could not only cause personal health problems [6] but also have a negative effect such as loss of work performance or decline in the quality of health care services [13,14]. As the COVID-19 pandemic has continued to linger into October 2021, identification of burnout among PHNs and associated factors could help enhance the professional quality of life of PHNs and could serve as important data for developing strategies to maintain the public health crisis management system.

Emotional labor is a stressor that leads to burnout [15], most PHNs live in the community where they work and provide public health nursing through direct interactions with the residents, and thus, they are even more vulnerable to stress and burnout caused by emotional labor [14,16]. Many previous studies have reported that excessive emotional labor can cause mental health problems, such as emotional distress [17], depression, anxiety, and frustration, as well as musculoskeletal symptoms, pain, menstrual irregularity [10], weakness, cardiovascular disease [18], and other physical health problems [19], while also causing a decrease in perceived health status (PHS). In a study that was conducted during the COVID-19 pandemic [5], PHNs felt physical and emotional fatigue and perceived their health status to be poorer due to face-to-face work for contact tracing, COVID-19-related tests, and self-isolation.

Higher emotional labor among nurses resulted in decreased job satisfaction [20], presenteeism, perceived organization support (POS) [21], and professional quality of life [16], which suggested that emotional labor negatively affects not only the individual but also the organization. POS refers to a set of beliefs that members have about organizational support, which includes such as organizational reward, fairness, favorable job conditions, working environments, closeness with colleagues, and positive atmosphere [22,23]. High POS reduces work stress, enhances resilience [24], increases job satisfaction and organizational commitment [25], and reduces burnout [11] among nurses. Other previous studies also reported that emotional labor [26] and POS [27] are factors affecting burnout of health care provider [28].

While there have been studies on emotional labor and burnout experienced by nurses under the recent COVID-19 pandemic, there is a lack of studies investigating the mediating factors in the association between emotional labor and burnout. Some previous studies on burnout among PHNs measured burnout status with just a single question; as a result, there is only limited information about burnout and there are difficulties in identifying its association with other factors. Accordingly, this study aimed to present basic data for developing burnout prevention programs by investigating the associations between emotional labor, PHS, POS, and burnout experienced by PHNs during the COVID-19 pandemic, which has over two years.

### 1.2. Purpose

The purpose of this study was to identify the mediating effects of PHS and POS in the association between emotional labor and burnout in PHNs. The specific purposes were: (1) to identify COVID-19 pandemic-related psychological burdens experienced by PHNs; (2) to identify the mediating effects of PHS and POS in the association between emotional labor and burnout in PHNs.

## 2. Methods

### 2.1. Study Design

This study was a cross-sectional descriptive survey using a structured questionnaire to investigate the associations between emotional labor, PHS, POS, and burnout perceived by PNHs during the COVID-19 pandemic.

### 2.2. Participants and Data Collection

The contents and methods of the study were approved by the Institutional Review Board of the university affiliated with the authors prior to the start of the study. Data were collected between February and March 2021. The participants were convenience sampled from PHNs with at least six months of work experience in public health centers and offices, graduated from 3-year or 4-year university in Jeju, Korea. At the time of the survey, there were six public health centers and 48 public health offices in Jeju Island, with 336 PHNs working in those institutions. Of these, permission for data collection was obtained from the heads of six public health centers and 24 public health offices. A single researcher personally visited the participants while following the government infectious disease control guidelines. The single researcher explained the overall content of the study (objective, survey method, questionnaire, to the candidates who satisfy the inclusion criteria by one to one. Ethical considerations were explained to the participants, that the collected data would only be used for the study and that there would be no disadvantages if they were to withdraw from the study. In addition, questionnaires were distributed to those who submitted a signed consent from one to one. The questionnaire, which required approximately 20 min to complete, was filled out by each participant and retrieved immediately upon completion on the spot. Each participant was given a small token of appreciation (approximately USD 10 in value). When the sample size needed for regression analysis was calculated using the G*Power 3.1.2 program (moderate effect size of 0.15, significance level of 0.05, and statistical power of 95%), the minimum sample size was 172. Considering the drop-out rate, a total of 210 candidates were recruited. After excluding three candidates with too many missing responses, data from a total of 207 participants were used in the final analysis.

The mean age of the participants was 37.4 years (range: 22–59 years), with 36.7% of the participants aged < 30 years. Of the participants, 96.1% were females, 55.1% had a spouse, and 46.9% were full-time workers. The percentage of participants with clinical nursing experience of ≥5 years was 51.2%, and the percentage of those with public health center work experience of ≥3 years was 56.5%. The percentage of participants who listed “visiting nursing” and “infectious disease control” as their main responsibility was 40.6% and 31.9%, respectively. The percentage of participants with <6 work hours per day for the past one month was 46.4%; 50.7% of participants had ≥4 h of COVID-19-related work experience; 63.8% had ≥2 h of COVID-19-related complaint response times; 59.9% had ≥2 h of overtime work (Table 1).

### 2.3. Measures

#### 2.3.1. General Characteristics of Participants

The general characteristics of participants surveyed included gender, age, spouse status, type of work institution (public health center/office), employment type (full time, part-time, and public service), clinical nursing experience (in years), work experience in public health center (in years), responsibilities (multiple responses possible), work hours per day for past one month, COVID-19-related work hours, COVID-19-related complaint response time, and overtime work.

#### 2.3.2. COVID-19 Pandemic-Related Psychological Burden

To identify psychological burden, the question, “how much psychological burden did you feel due to the COVID-19 pandemic” in past one month was asked for five items. Each item was graded on a scale of “not difficult at all” (1 point) to “very difficult” (10 points), with higher total scores indicating higher psychological burden. The validity of the tool was assessed by two nursing professors and three PHNs with more than 10 years of experience, and 5 items with a content validity index (CVI) of 0.8 or higher were selected. The overall CVI was 0.9.

#### 2.3.3. Burnout

Burnout was measured using the Korean version of the tool developed by Pines et al. [29]. This tool consists of a total of 21 items, including physical burnout (7 items), emotional burnout (7 items), and mental exhaustion (7 items). Each item is graded on a 5-point Likert scale (not at all = 1 to always so = 5), with higher total scores indicating a higher level of burnout. In this study, the reliability of the tool was Cronbach’s α = 0.92.

#### 2.3.4. Emotional Labor

Emotional labor was measured using the Korean version of the tool developed by Morris and Feldman [30]. This tool consists of a total of 9 items, including frequency of emotional labor (3 items), attention level of emotional expression (3 items), and emotional dissonance (3 items). Each item is graded on a 5-point Likert scale (Not at all = 1 to Very much so = 5), with higher total scores indicating higher emotional labor intensity. In this study, the reliability of the tool was Cronbach’s α = 0.88.

#### 2.3.5. PHS

PHS was measured using the Korean version of the tool developed by Speake et al. [31]. This tool consists of a total of 3 items: current overall health status, health status compared with peers, and health status satisfaction compared with one years ago. Each item is graded on a 5-point Likert scale (Not at all = 1 to Very much so = 5), with higher total scores indicating better PHS. In this study, the reliability of the tool was Cronbach’s α = 0.89.

#### 2.3.6. POS

POS was measured using the Korean version of the tool developed by Eisenberger and Huntington [22]. This tool consists of a total of 8 items, including items such as ‘our organization values my contribution’. Each item is graded on a 7-point Likert scale (Not at all = 1 to Very much so = 7), with higher total scores indicating higher POS. In this study, the reliability of the tool was Cronbach’s α = 0.88.

### 2.4. Data Analysis

Collected data were analyzed using SAS 9.4 program. The general characteristics of participants, emotional labor, PHS, POS, and burnout were analyzed using descriptive statistics. Correlations were verified by Pearson’s correlation coefficients. To verify the mediating effects of PHS and POS in the association between emotional labor and burnout, PROCESS macro-SPSS/WIN 3.3 program was used to analyze by a parallel multiple mediator model [32] while bootstrapping was used for inference of indirect effect. Emotional labor was inputted as the independent variable; burnout as the dependent variable; PHS and POS as mediating variables. Analysis was performed by inputting four (parallel multiple mediator model) as the model number, 95% confidence interval (CI), and 10,000 as the bootstrap sample size. Before testing the mediating effects, multicollinearity between independent variables and autocorrelation between dependent variables were checked. The suitability of the regression model was checked by a normal distribution (Kolmogorov–Smirnov’s) and homoscedasticity (Breusch–Pagan’s) using residual analysis.

## 3. Results

### 3.1. Psychological Burden, Burnout, and Related Factors

The psychological burden due to the COVID-19 pandemic appeared in the order of “Being stricter on one’s self than as required by the government with respect to social distancing and restrictions on personal life” (7.36 out of 10 points), “overload of various work assigned additionally because of being a nurse” (7.09 points), and “social expectations about sacrifice as a public servant and expectation of kind response” (6.84 points). The mean score of emotional labor was 31.55 out of 45 points, the mean score of PHS was 9.09 out of 15 points, the mean score of POS was 32.82 out of 56 points, and the mean score of burnout was 63.29 out of 105 points (Table 2).

### 3.2. Correlations between Burnout and Related Factors

Burnout was positively correlated with emotional labor (r = 0.64, *p* < 0.001) and negatively correlated with PHS (r = −0.51, *p* < 0.001) and POS (r = −0.51, *p* < 0.001). Emotional labor was negatively correlated with PHS (r = −0.42, *p* < 0.001) and POS (r = −0.41, *p* < 0.001). PHS was positively correlated with POS (r = 0.38, *p* < 0.001) (Table 3).

### 3.3. Mediating Effects of PHS and POS in the Association between Emotional Labor and Burnout

Before analyzing the mediating effects of PHS and POS in the association between emotional labor and burnout in PHNs, multicollinearity between independent variables was checked. It was determined that there is no multicollinearity between independent variables based on tolerance ≥ 0.1 (0.75–0.78), variance inflation factor (VIF) < 10 (1.29–1.33), and correlation coefficient < 0.80 (0.38–0.64). Moreover, the Durbin–Watson value was close to 2.00 with 1.75, indicating no problem with the autocorrelation of errors. With respect to the suitability of the regression model for burnout, Kolmogorov–Smirnov residual normality test results showed Z = 0.05 and *p* = 0.200 (>0.05) to satisfy the assumption of residual normality, while Breusch–Pagan homoscedasticity test results showed *p* = 1.000 (>0.05) to satisfy the assumption of homoscedasticity, which confirmed the suitability of the regression model.

The parallel multiple mediator model was analyzed using Process macro V.3.3, which is capable of simultaneously testing both direct and mediating effects using regression analysis. The independent variable emotional labor had a significant effect on mediating variables PHS (B = −0.17, *p* < 0.001) and POS (B = −0.53, *p* < 0.001), while the independent variable emotional labor (B = 1.07, *p* < 0.001) and mediating variables PHS (B = −1.36, *p* < 0.001) and POS (B = −0.42, *p* = 0.001) all had a significant effect on the dependent variable burnout.

**Figure 1:**
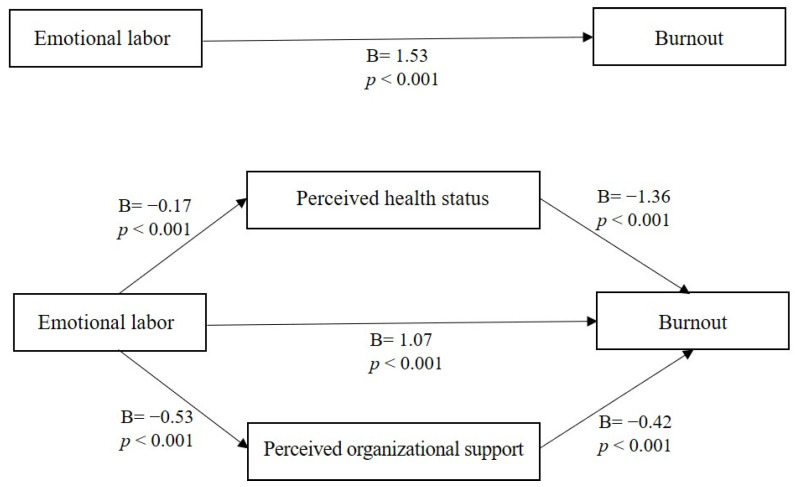
Multiple mediation bootstrap analysis of relationships between emotional labor and burnout as mediated by perceived health status and perceived organizational support.

The direct effect size of emotional labor on burnout was 1.07, and the results were significant since 95% bootstrap CI did not include 0 (0.81–1.33). The indirect effect size of emotional labor on burnout mediated by PHS was 0.24; the results were statistically significant since 95% bootstrap CI did not include 0 (0.11–0.39), while the indirect effect size of emotional labor on burnout mediated by POS was 0.23. The results were significant since 95% bootstrap CI did not include 0 (0.07–0.42). The sum of indirect effects was 0.46, and the results were significant since 95% bootstrap CI did not include 0 (0.29–0.68). These results indicated that higher emotional labor perceived by PHNs could reduce PHS and POS to increase burnout (Table 4, Figure 1).

## 4. Discussion

This study aimed to present basic data for developing an intervention for reducing or preventing burnout due to emotional labor by identifying the level of psychological burden and investigating the mediating effect of PHS and POS in the associations between emotional labor and burnout in PHNs during the ongoing COVID-19 pandemic.

The findings in the study showed that “being stricter on one’s self than as required by the government with respect to social distancing and restrictions on personal life” was the highest among the psychological burdens experienced by PHNs, followed in order by “Overload of various work assigned additionally because of being a nurse” and “social expectations about sacrifice as a public servant and expectation of kind response.” While the direct comparison is difficult due to the lack of previous studies on PHNs, the findings were consistent with a Q-methodology study on public hospital nurses in Korea [33], which reported that nurses experienced psychological stress due to restrictions on the private life of not only themselves but also their family, fear of social stigma when exposed to infection, and excessive workload. Another study on public hospital nurses in Turkey [34] reported that nurses experienced psychological difficulties due to self-imposed restrictions on social activities, loneliness, occupational role conflict, and organizational expectation for crisis management.

During the COVID-19 pandemic, PHNs were initially mobilized for infection control and quarantine, while actively adapting to constantly changing policies and guidelines. They felt pressure from still being responsible for the regular role of providing health promotion and public health services, such as smoking cessation education and vaccination [1] while facing additional COVID-19-related work [35]. In this study, over 50% of PHNs had a heavy work burden, having performed ≥ 4 h of COVID-19-related work (50.7%), ≥2 h of complaint response work (63.8%), and ≥2 h of overtime work (59.9%) in the past one week. Based on such findings, it was determined that under the COVID-19 pandemic, PHNs felt burden from social responsibilities and obligations and role expectations from the community and organization; because nurses are also public servants, their sense of responsibility was doubled. Moreover, they were being overburdened due to the addition of COVID-19-related work, in addition to their regular work.

The first finding in this study showed that higher emotional labor perceived by PHNs directly increased burnout. Various previous studies have already reported the negative effect of emotional labor on burnout among nurses [15,19,36]. In a qualitative study on nurses by Lee and Lee [8], nurses struggled with professional responsibilities despite mounting fatigue and added workload due to the non-ending pandemic, but they felt emotional burnout from dealing with non-cooperative patients who repeatedly violate infection control rules, which supported the findings in this study. In a previous study, emotional labor in the domain of “emotional dissonance and hurt” had most effect on burnout [37] and PHNs may experience emotional hurt due to emotional dissonance when they repeatedly show only the emotions demanded by the organization for successful complaint response while suppressing their own emotions. Meier et al. [38] mentioned that senior managers, organizations, and outsiders tend to think that “suppressing one’s own emotions and expressing the emotions the organization wants even in situations with increased emotional labor is the price of labor for civil servants,” which suggested that response to emotional labor may be slow among civil servants. Therefore, it is necessary to recognize one’s own emotions and learn effective emotional management and proper emotional expression methods to manage injury caused by emotional labor.

The second finding in this study showed that PHS has a partial mediating effect on the association between emotional labor and burnout in PHNs. In previous studies, the excessive emotional labor of health care professionals was reportedly associated with physical and mental health problems, such as somatic symptoms [39], depression, hypertension, and heart disease [18]; deterioration of health, such as persistent fatigue, gastrointestinal problem, cardiovascular disorder, and diabetes, could be predictors of burnout among office workers [12]. Moreover, other studies reported that lower subjective health status among PHNs is associated with a higher perceived level of burnout [14]; health care professionals with health problems are more vulnerable to personal and work-related burnout [5]. Such results suggested that long-term accumulation of emotional labor can cause deterioration of personal health [10] to cause an increase in burnout.

The results in this study showed that higher emotional labor in PHNs is associated with a decrease in PHS, which leads to an increase in burnout. It suggests that burnout could be relieved by positively responding to the negative effect of emotional labor if the PHS of PHNs can be enhanced. This study surveyed the PHS of PHNs. However, mental stress may manifest as physical symptoms before it is perceived [40]; thus, PHNs must consistently put forth the efforts for early response and continued self-health care once abnormal health symptoms are perceived. Organizations need to put forth efforts to establish a mutually supportive network within public health centers to create an open and healthy workplace culture.

The third finding in this study showed that POS has a partial mediating effect on the association between emotional labor and burnout in PHNs. In other words, higher emotional labor is associated with a decrease in POS, which is in turn associated an increase in burnout. Previous studies reported a negative association between emotional labor and POS among nurses [21] and that when nurses perceived that the organization lacked consideration for the importance of employees and their well-being, there was a decrease in their professional self-concept [11], job commitment, and job satisfaction [24,25]. In contrast, the perception of a pleasant working environment and a sense of receiving organizational care could reduce work-related burnout [28]. These results supported the findings in this study. In a study that included nurses and midwives, more surface acting due to emotional labor was associated with a decrease in POS, which led to decreased job satisfaction [25]. Lartery et al. [25] reported that positive organizational resources could act as a protective factor against the negative consequences of these emotional requirements.

Labrague and de los Santos [41] reported that higher POS among nurses is associated with lower COVID-19-related anxiety while proposing that appropriate organizational support (e.g., structural support, effective communication, safe working environment, COVID-19-related education, and well-being monitoring) is essential for supporting nurses who are directly facing difficulties due to the COVID-19 crisis.

The findings in this study suggested that PHNs need organizational support that they can empathize with when managing emotional labor during their work, which could help alleviate burnout. When PHNs perceive that their own organization will provide even more support, their own emotional regulations could have a positive effect on the prevention of burnout [28]. The Korean government and health authorities were lauded for having an effective disease control by supporting a “K-Quarantine Model” with support of public health personnel and resources, the establishment of an online healthcare system, testing-contact tracing-quarantine through campaigns, and treatments to prevent the spread of COVID-19 in the early stage of the pandemic [42]. However, stable and systematic support has not been easy due to the ongoing pandemic trend, vaccine supply and demand, vaccination, sporadic nationwide outbreaks, and virus mutation. Nonetheless, the roles of PHNs are expected to expand even more since the COVID-19 pandemic is still ongoing, and time has come to implement policies for “with COVID”; thus, organizational and government support for improving working conditions is essential and urgently needed.

These findings determined that higher emotional labor perceived by PHNs could lead to a decrease in PHS and POS, which is associated with an increase in burnout. This suggested that when PHNs experience high emotional labor, increasing PHS and strengthening POS could help alleviate burnout. Interventions that provide effective emotional regulation management education, communication, and psychological counseling support [43] to positively manage the negative effects of emotional labor and maintain fitness and health by recommending meditation, yoga, or exercise [44] could help reduce or prevent PHNs’ burnout. Moreover, managers and organizations where the PHNs belong should analyze the COVID-19-related burden, role change, and work scope to provide organizational support, such as adequate rest, flexible work hours, work allocation, and appropriate personnel management, to alleviate burnout [9].

This study had some limitations. Firstly, there are limitations in generalizing the findings since the study population consisted of PHNs from just one region in Korea. Secondly, because health status was identified based on subjective health status perceived by PHNs, it is necessary to check the actual physical health problems. Thirdly, this study did not consider actual organizational support, such as pay, benefits, and working environment. Future studies should investigate organizational support for PHNs during the COVID-19 pandemic and its outcomes. Fourthly, because the psychological burden was surveyed with a single question, there are limitations in interpreting the results; the causal relationship with emotional labor or burnout could not be identified. There is a need to conduct in-depth qualitative studies on the effects on psychological burden and burnout with consideration for the job characteristics of PHNs. Moreover, this study did not identify the mediating effects of work engagement [45], stress [39], and job satisfaction [16], which could affect the association between emotional labor and burnout in PHNs.

Because PHS and POS, in relation to emotional labor and burnout, were investigated by a cross-sectional survey, there are limitations in generalizing the findings; in the future, there is a need for longitudinal design studies on the accumulation of fatigue in PHNs and identification of outcomes regarding burnout due to the continuation of the pandemic.

This study has limitations in generalizing the results because convenience sampling was used to obtain participants. Despite these limitations, this study was significant in that it presented measures for reducing burnout in PHNs by testing the mediating effects of PHS and POS in the association between emotional labor and burnout in PHNs responsible for providing public health services during the COVID-19 pandemic.

## 5. Conclusions

This study analyzed the mediating effects of PHS and POS in how the emotional labor of PHNs affect burnout to provide basic data for the development of burnout prevention programs for nurses who play a central role in public healthcare service, while also presenting the need for interventions at personal and organizational levels. It presented evidence that when emotional labor is high, increasing PHS and strengthening POS could help reduce burnout.

Based on these findings, it is believed, to reduce burnout among PHNs, it is necessary to develop and operate burnout prevention programs to increase PHS and strengthen organizational support while also reducing emotional labor through physical activities and emotional regulation. Future studies are recommended for investigating work-related burnout among PHNs and physical and emotional mediating or regulating variables that may have changed after the COVID-19 pandemic.

## Figures and Tables

**Table 1:** Burnout according to general characteristics of participants.

Characteristics	Categories	N (%) or M ± SD	Burnout	
			M ± SD	t or F(*p*) Scheffe Test
Age		37.4 ± 11.0 (22–59)		
	<30	76 (36.7)	66.12 ± 14.36	5.44 (0.001)
	30~39	51 (24.6)	65.12 ± 14.29	a, b, c > d
	40~49	38 (18.4)	62.82 ± 12.10	
	≥50	42 (20.3)	56.36 ± 9.67	
Gender	Female	199 (96.1)	63.79 ± 13.18	−2.71 (0.007)
	male	8 (3.9)	50.75 ± 17.11	
Spouse	No	93 (44.9)	65.27 ± 13.71	1.92 (0.057)
	Yes	114 (55.1)	61.67 ± 13.23	
Working institution	Public health care center	181 (87.4)	63.79 ± 13.59	1.42 (0.157)
	Public health office	26 (12.6)	59.77 ± 12.81	
Employment type	Regular worker	97 (46.9)	64.84 ± 11.50	3.49 (<0.001)
	Irregular workers	110 (53.1)	59.19 ± 11.70	
Total nurse career (years)	<5	101 (48.8)	64.18 ± 13.90	1.11 (0.600)
	≥5	106 (51.2)	62.43 ± 13.19	
Career of employment in healthcare center (year)	<3	90 (43.5)	65.01 ± 14.01	1.62 (0.108)
	≥3	117 (56.5)	61.96 ± 13.06	
Responsibilities *	Visiting nursing	84 (40.6)		
	Infectious disease control	66 (31.9)		
	Health promotion	34 (16.4)		
	Vaccination	31 (15.0)		
	Health administration	26 (12.6)		
	Medical management	7 (3.4)		
	Mental health	12 (5.8)		
	Maternal and child health	17(8.2)		
	Others	37 (17.9)		
Responsible business hours, Median (range)		5.94 ± 3.01 (0–15)		
	<6	96 (46.4)	65.25 ± 11.73	1.99 (0.048)
	≥6	111 (53.6)	61.59 ± 14.76	
COVID-19 related business hours, Median (range)		4 (0–32)		
	<4	102 (48.3)	60.07 ± 12.14	−3.46 (<0.001)
	≥4	105 (50.7)	66.14 ± 14.13	
COVID-19 related complaints response time, Median (range)		2 (0–55)		
	<2	75 (36.2)	58.31 ± 14.17	−4.14 (<0.001)
	≥2	132 (63.8)	66.11 ± 12.34	
Overtimes		2 (0–24)		
	<2	83 (40.1)	58.99 ± 13.88	−3.86 (<0.001)
	≥2	124 (59.9)	66.16 ± 12.55	

* Multiple response.

**Table 2:** Psychological burden, burnout, emotional labor, perceived health status, perceived organizational support.

Variables	M ± SD	Min-Max
COVID-19 pandemic related to psychological burden		
Being stricter on one’s self than as required by the government with respect to social distancing and restrictions on personal life	7.36 ± 2.37	0–10
Overload of various work assigned additionally because of being a nurse	7.09 ± 2.60	0–10
Social expectations about sacrifice as a public servant and expectation of kind response	6.84 ± 2.62	0–10
Verbal and physical abuse from complaints	6.06 ± 2.81	0–10
Daily life of family members being restricted due to me	6.65 ± 2.71	0–10
Emotional labor	31.55 ± 5.68	16–45
Perceived health status	9.09 ± 2.35	3–15
Perceived organizational support	32.82 ± 7.33	8–55
Burnout	63.29 ± 13.53	31–97

**Table 3:** Correlation among emotional labor, perceived health status, perceived organizational support and burnout.

Variables	Emotional Labor	Perceived Health Status	Perceived Organizational Support	Burnout
	r (*p*)	r (*p*)	r (*p*)	r (*p*)
Emotional labor	1			
Perceived health status	−0.42 (<0.001)	1		
Perceived organizational support	−0.41 (<0.001)	0.38 (<0.001)	1	
Burnout	0.64 (<0.001)	−0.51 (<0.001)	−0.51 (<0.001)	1

**Table 4:** Effects of public health nurses’ emotional labor and burnout: The mediating effects of perceived health status and perceived organizational support.

Variables	B	SE	t	*p*	95% CI			
Emotional labor→Perceived health status	−0.17	0.03	−6.63	<0.001	−0.23~−0.12			
Emotional labor→Perceived organizational support	−0.53	0.08	−6.49	<0.001	−0.69~0.37			
Emotional labor→Burnout	1.07	0.13	8.03	<0.001	0.81~1.33			
Perceived health status→Burnout	−1.36	0.32	−4.31	<0.001	−1.99~−0.74			
Perceived organizational support→Burnout	−0.42	0.10	−4.18	<0.001	−0.62~−0.22			
**Variables**	**Directing effect**				**Indirect effect**			
	**B**	**Boot SE**	**95% CI**		**B**	**Boot SE**	**95% CI**	
			**Boot LLCI**	**Boot ULCI**			**Boot LLCI**	**Boot ULCI**
Emotional labor→Burnout	1.07	0.13	0.81	1.33				
Emotional labor →Perceived health status→Burnout					0.24	0.07	0.11	0.39
Emotional labor→Perceived organizational support→Burnout					0.23	0.09	0.07	0.42
Total					0.46	0.10	0.29	0.68

CI = confidential interval.

## Data Availability

Data are available upon request.
